# The unlikely suspect: A case of isolated pulmonary mucormycosis-induced acute mediastinitis in an immunocompetent patient

**DOI:** 10.5339/qmj.2024.qitc.22

**Published:** 2024-04-30

**Authors:** Mustafa A. Al-Tikrity, Khaled Alsa’ed, Ahmed N. Elgohari, Nadir Kharma, Mansoor Hameed

**Affiliations:** 1 Hamad General Hospital, Hamad Medical Corporation, Doha, Qatar Email: maltikrity@hamad.qa; 2 Weill Cornell Medicine – Qatar, Cornell University, Doha, Qatar

**Keywords:** Fungal infection, Mucormycosis, Pulmonary, Acute mediastinitis, Diabetes

## Background

Mucormycosis is a highly lethal invasive fungal infection usually found in immunocompromised patients. An increasing incidence is seen in uncontrolled diabetes.^1^ Pulmonary involvement can manifest as an acute or chronic disease and may present with infiltrates, consolidation, or cavities.^2^

## Case Presentation

**Figure 1.**
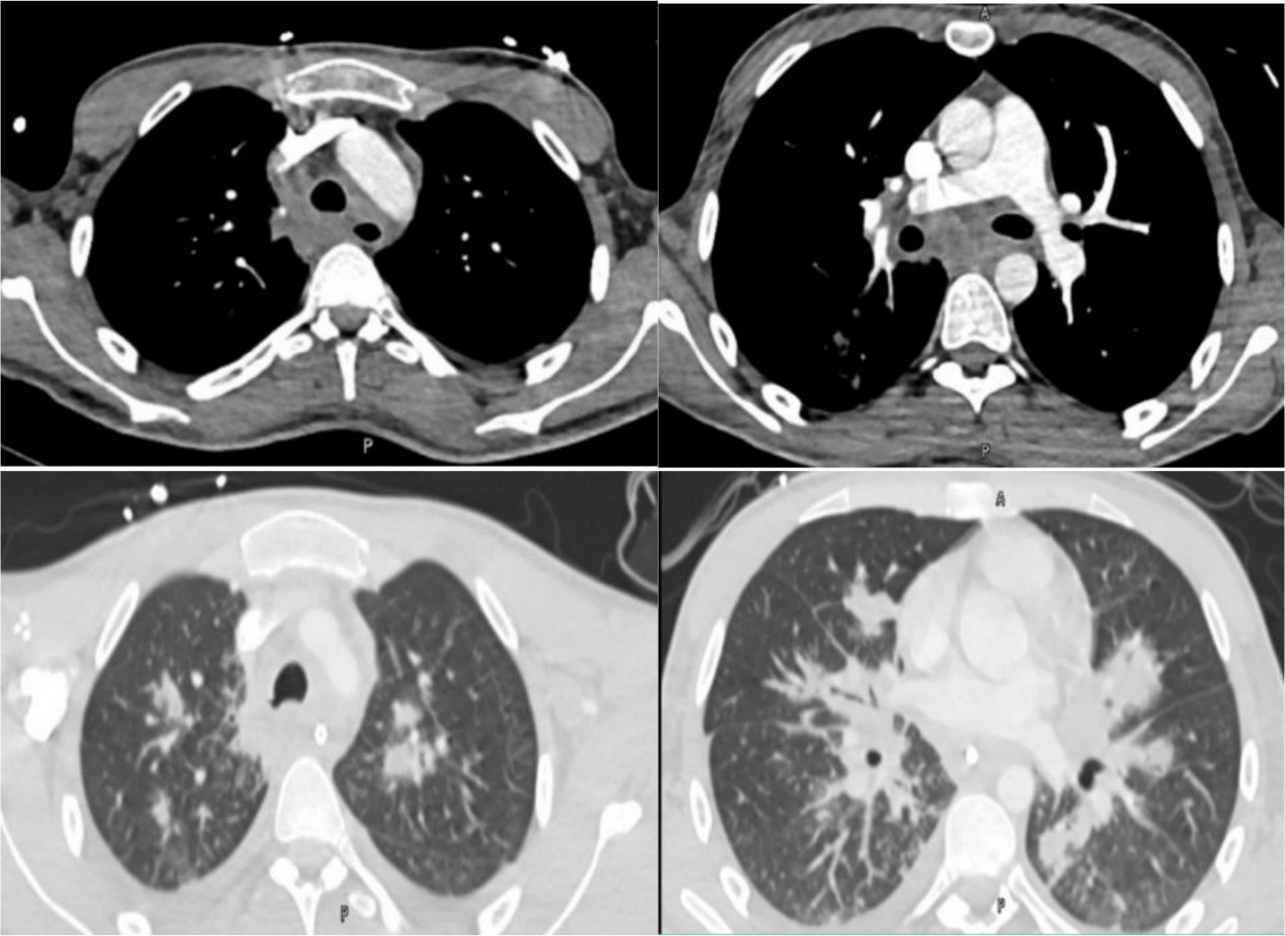
CT pulmonary angiogram shows extensive infiltrative changes in the mediastinum, and lung consolidations suggestive of acute mediastinitis and lung involvement.

**Figure 2.**
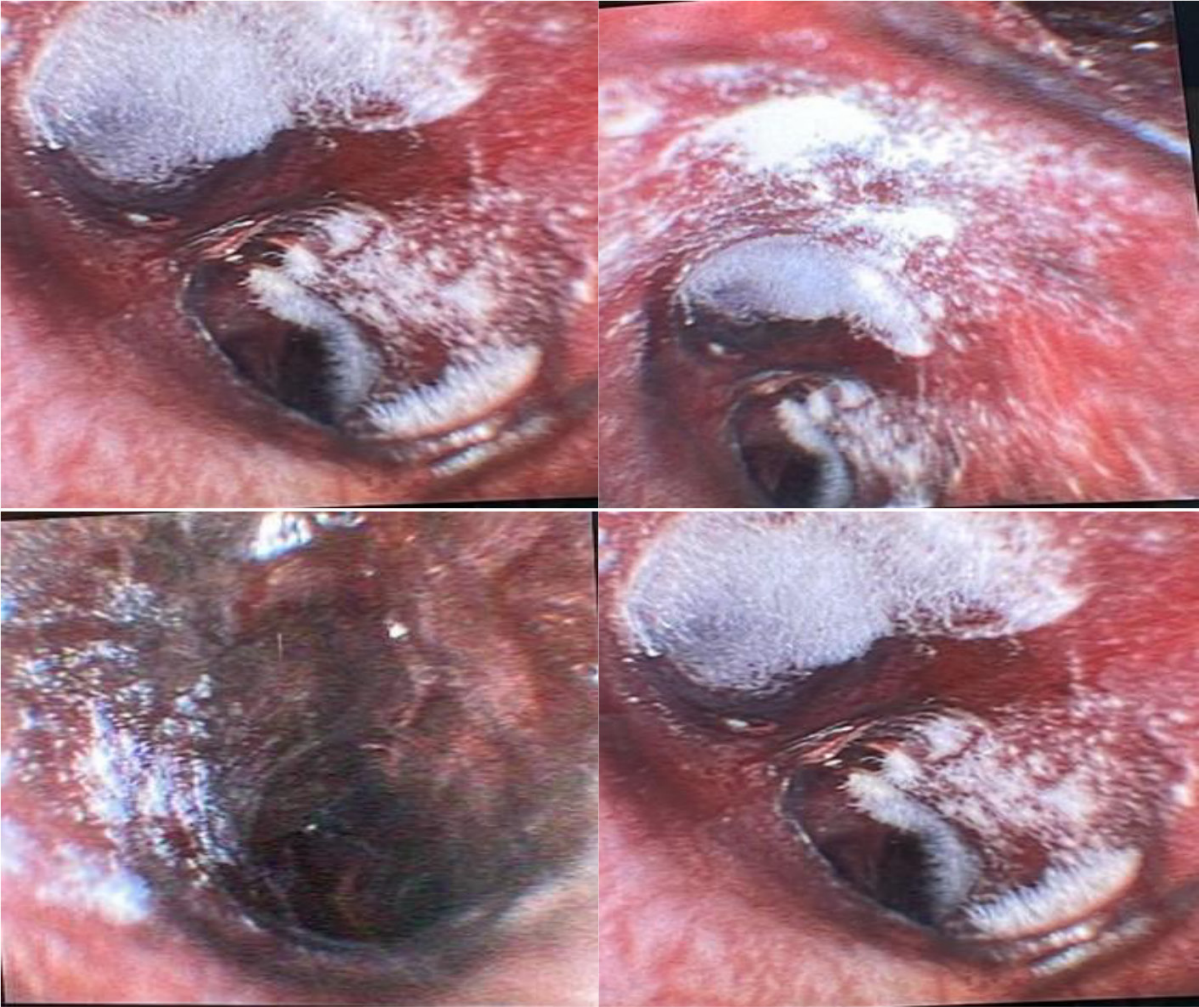
Images show severe tracheobronchitis with pseudo-membrane and necrosis.

A 20-year-old male with type 1 diabetes mellitus, who was non-compliant with his medications, presented to the hospital with shortness of breath, productive cough, sore throat, and fever for over one week. He had ketone breath and a clear chest on auscultation. Initial tests revealed severe metabolic acidosis with ketones, and treatment for diabetic acidosis was initiated. A CT scan of the chest showed extensive infiltrative changes in the mediastinum, and lung infiltrates were suggestive of acute mediastinitis and lung involvement (Figure 1). Due to respiratory distress, the patient was intubated and subsequently underwent bronchoscopy, which revealed severe tracheobronchitis and pseudo-membrane with necrosis (Figure 2). Bronchoalveolar lavage showed Rhizopus. A CT scan of the neck showed small fluid collections in various compartments. Initially, the patient received antibiotics and after confirmation of the fungal infection, high-dose amphotericin and caspofungin were added to the therapeutic regimen. After initial extubation, the patient had to be re-intubated due to desaturation. The patient’s clinical course was marred by recurrent fever, desaturations, and respiratory acidosis. A follow-up CT scan of the neck and chest three weeks later showed disease progression with new cystic and varicoid bronchiectasis and extensive consolidations. Five weeks after admission, his condition deteriorated further, and he unfortunately died of refractory sepsis.

## Conclusion

This case report highlights the complex clinical course of a diabetic patient with acute severe mediastinitis, tracheobronchitis, and lung involvement due to mucormycosis. Obtaining histopathological and microbial evidence through aggressive performance of invasive procedures is crucial for a definitive and timely diagnosis of this highly fatal disease, which lacks specific clinical and imaging manifestations, and to prevent misdiagnosis and delayed treatment.

## Conflict of Interest

The authors declare no conflict of interest regarding this case report.

## References

[1] Binder U, Maurer E, Lass-Flörl C (2014;). Mucormycosis - From the pathogens to the disease. Clinical Microbiology and Infection.

[2] Agrawal R, Yeldandi A, Savas H, Parekh ND, Lombardi PJ, Hart EM (2020;). Pulmonary mucormycosis: Risk factors, radiologic findings, and pathologic correlation. Radiographics.

